# Something old or something new? Social health insurance in Ghana

**DOI:** 10.1186/1472-698X-9-20

**Published:** 2009-08-28

**Authors:** Sophie Witter, Bertha Garshong

**Affiliations:** 1 Immpact, Institute of Applied Health Sciences, University of Aberdeen, Aberdeen, UK; 2 Health Research and Development Division, Ghana Health Services, Accra, Ghana

## Abstract

**Background:**

There is considerable interest at present in exploring the potential of social health insurance to increase access to and affordability of health care in Africa. A number of countries are currently experimenting with different approaches. Ghana's National Health Insurance Scheme (NHIS) was passed into law in 2003 but fully implemented from late 2005. It has already reached impressive coverage levels. This article aims to provide a preliminary assessment of the NHIS to date. This can inform the development of the NHIS itself but also other innovations in the region.

**Methods:**

This article is based on analysis of routine data, on secondary literature and on key informant interviews conducted by the authors with stakeholders at national, regional and district levels over the period of 2005 to 2009.

**Results:**

In relation to its financing sources, the NHIS is heavily reliant on tax funding for 70–75% of its revenue. This has permitted quick expansion of coverage, partly through the inclusion of large exempted population groups. Card holders increased from 7% of the population in 2005 to 45% in 2008. However, only around a third of these are contributing to the scheme financially. This presents a sustainability problem, in that revenue is de-coupled from the growing membership. In addition, the NHIS offers a broad benefits package, with no co-payments and limited gate-keeping, and also faces cost escalation related to its new payment system and the growing utilisation of members. These features contributed to a growth in distressed schemes and failure to pay outstanding facility claims in 2008.

The NHIS has had a considerable impact on the health system as a whole, taking on a growing role in funding curative care. In 2009, it is expected to contribute 41% of the overall resource envelope. However there is evidence that this funding is not additional but has been switched from other funding channels. There are some equity concerns about this, as the new funding source (a VAT-based tax) may be more regressive. In addition, membership of the NHIS at present has a pro-rich bias, and a pro-urban bias in relation to renewals. Only a very small proportion is registered as indigent, and there is some evidence of 'squeezing out' of non-members from health care utilisation. Finally, considerable challenges remain in relation to strengthening the purchasing role of the NHIS, and also settling debates about its structure and accountability.

**Conclusion:**

Some trade-offs will be necessary between the existing wide benefits package of the NHIS and the laudable desire to reach universal coverage. The overall resource envelope for health is likely to be stable rather than increasing over the medium-term. In the longer term, the investment costs in the NHIS will only be justified if it is able to increase the cost-effectiveness of purchasing and the responsiveness of the system as a whole.

## Background

In the light of the Millennium Development Goal targets for health gains and poverty reductions, there is a growing impetus towards providing universal coverage of health services [1], meaning that all of the population has access to appropriate health care when needed, and at an affordable cost. One important measure to increase affordability is to reduce the out-of-pocket payments which users make for health care. These are widely recognised as creating a barrier to access, especially in poorer countries, and as pushing households further into poverty [2].

Social health insurance is seen as one of the health financing approaches with a strong potential to share risks across population groups and time. As membership is mandatory, it avoids many of the problems of adverse selection which smaller, voluntary health insurance schemes face. Until recently, there were few large-scale social health insurance schemes operating in sub-Saharan Africa (ones which extended beyond smaller groups of formal sector workers, such as civil servants). Over the past decade, however, experiments in social health insurance have been springing up in a number of African countries, including Nigeria, Rwanda, Kenya, Tanzania, and Ghana [3]. As these schemes are still young and evolving, few have yet been systematically evaluated.

The National Health Insurance Act (Act 650) was passed into law in Ghana in 2003, though implementation (in terms of access to benefits) began in autumn of 2005. At four years old, it is timely to assess its evolution and performance to date. This article examines the design of the National Health Insurance Scheme (NHIS) in Ghana and the evidence to date of its impact, both on members and on the health system as a whole. The NHIS has seen a rapid increase in membership during the first four years of its life. We examine how well is it performing against its overall objectives, and what lessons it can provide for the region.

The NHIS in Ghana grew out of an election promise made in 2000 by the incoming New Patriotic Party to abolish user fees (traditionally known in Ghana as 'cash and carry') [4]. These have constituted a well-documented barrier to health care in Ghana since the 1980s, and attempts to alleviate them with a system of exemptions have not been very successful [5-7]. As a proportion of total public sector funding, user fees constituted 13–14% in 2005 [8]. Act 650 and the subsequent Legislative Instrument (LI 1809) of 2004 do not specify the goals of the policy, but the original focus from the party manifesto was clearly on removing financial barriers to utilisation of health care.

During the 1990s, a number of mutual health organisations (MHOs) developed in Ghana, with some external funding and technical support. Most MHOs focussed on providing financial protection against the potentially catastrophic costs of a limited range of inpatient services [9]. The NHIS aimed to build on these organisations by introducing district-based mutual health insurance schemes (DMHIS).

The main features of the NHIS in Ghana are summarised in Table 1. It was designed as a mandatory health insurance system, with risk pooling across district schemes, funded from members' contributions and a levy on the value-added tax (VAT) charged on goods and services, from which a broad minimum package of care could be funded.

**Table 1:** Main features of Ghana NHIS

Feature	Description
Funding	National Health Insurance Fund (NHIF) established to pay for:
	▪ Subsidies to schemes
	▪ Reinsurance for schemes
	▪ Cost of enrolling the indigent
	▪ Supporting access to health care
	
	Funds to come from:
	▪ National Health Insurance Levy (NHIL) – 2.5% of V.A.T.
	▪ Payroll deductions (2.5% of income) for formal sector
	▪ employees
	▪ Other funds voted by Parliament, income from investments, any donations, or loans
	
	In addition, DHMIS will raise funds from premia for informal sector members, to be set by agreement with the National Health Insurance Authority (NHIA)

Membership	Membership is mandatory (either via the DHMIS or a private insurance policy). Formal sector workers have involuntary payroll deductions (SSNIT contributions). Informal sector are charged premia which should be income-related. Initially, there is a six-month gap between joining and being eligible for benefits.

Exemptions	Some groups will be exempt from paying for membership (originally SSNIT pensioners, over-70s, under-18s where both parents are members; indigents). The NHIA will transfer subsidies to cover the cost of their enrolment. An indigent is defined as someone who meets four criteria:
	▪ is unemployed and has no visible source of income;
	▪ does not have a fixed place of residence according to standards determined by the scheme;
	▪ does not live with a person who is employed and who has a fixed place of residence; and
	▪ does not have any identifiable consistent support from another person.

Benefits package	All providers must offer a minimum package, which is specified and broad. National Health Insurance Drug List is established. 95% of all health care is covered – all services are included other than: rehabilitation other than physiotherapy; appliances and prostheses; cosmetic surgery; HIV retroviral drugs; assisted reproduction; echocardiography; photography; angiography; orthoptics; kidney dialysis; heart and brain surgery other than those resulting from accidents; cancer treatment other than cervical and breast cancer; organ transplantation; non-listed drugs; treatment abroad; medical examinations for visas etc.; VIP wards; and mortuary services.

Eligible providers	All providers are eligible, once accredited. Accreditation is reviewed every five years. Quarterly reports to be sent to the NHIC by providers. Providers are to be paid within four weeks of claim being made to DMHIS.

Organisation	National Health Insurance Authority (NHIA) established to regulate the market, including accreditation of providers, agreeing contribution rates with schemes, resolving disputes, managing the NHIF, and approving cards. Each district to have a DMHIS (with a minimum of 2,000 members). Benefits to be transferable across district schemes. Each DHMIS to submit annual reports to NHIA and to undertake annual audit of accounts. Private MHIS not eligible for subsidies from NHIA.

Accountability	National Health Insurance Council (NHIC) established to oversee NHIA and licence schemes (every two years). Includes representatives of main stakeholder groups, such as Ministry of Health, Ghana Health Services, regulatory bodies, consumers, and Executive Secretary of the NHIA. Chair and Executive Secretary appointed by the President. NHIC proposes formula for allocation of funds to Parliament for annual approval, and provides annual report to Parliament on its use of funds. Each DHMIS governed by a Board. Rules established for handling complaints against providers or schemes.

*Source*: summarised from Act 650 (2003) and LI 1809 (2004)

## Methods

This article is based mainly on analysis of routine data reported in the Government of Ghana Budget Statements, Ministry of Health Financial Statements, NHIA reports, and annual health sector reviews. The chief comparison is between the situation in 2006 – the first full year of operation of the NHIS – and 2008 – the latest year for which some data are available. The data sources are analysed thematically, focussing on all of the main areas of concern for insurance schemes – coverage levels, revenues, cost components, and expenditures.

This is supplemented by two sources: a review of published and grey literature on the NHIS, and insights derived from semi-structured key informant interviews. These were conducted as part of annual health sector reviews in 2006, 2007 and 2009. The questions focussed on the implementation of the NHIS, its impact on key stakeholders and its sustainability. Informants were chosen purposively based on their positions and knowledge. They included representatives of the Ministry of Health, the Ministry of Finance, the NHIA, the Ghana Health Service, district schemes, development partners, non-governmental organisations, health facilities, and health managers at regional and district levels. In 2006 and 2007, 19 key informants were interviewed; in 2009 the number was 35.

## Results

### Funding sources

For most social health insurance (SHI) models, the principal source of funding is earmarked contributions by employees and their employers, civil servants and government (as their employer), and the self-employed from the formal and informal sectors of the economy. Hybrid forms of SHI are also quite prevalent, with government paying contributions for those, such as the unemployed and the poor, who would otherwise have difficulties in contributing. However, its distinct feature is that it does not call exclusively on public finance, but instead spreads the responsibility of health care financing among households and the private sector as well [3].

In the case of Ghana, the NHIA is predominantly financed from taxation (the hypothecated NHIL, described in Table 1), which constitutes an estimated 70–75% of total revenue, with a further 20–25% coming from formal sector contributions and only around 5% from informal sector premia (according to NHIA financial reports). This makes it less distinctly different from the traditional funding mechanisms (government budgets, donor funding and user fees), at least in terms of revenue generation. This situation may be exacerbated if, as promised during its election manifesto, the current NDC government shifts to a 'one-time payment' conferring life membership (presumably for the informal sector alone). This will further erode the notion that the NHIS is a contribution-based insurance system.

### Coverage

**Figure 1:**
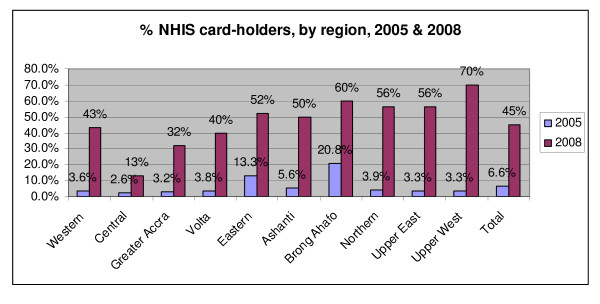
**Proportion of NHIS card-holders, by region, 2005 & 2008**. *Source*: analysis of data presented in annual health sector reviews for 2005 and 2008.

Membership of the NHIS is legally mandatory (unless alternative private health insurance can be demonstrated); however, in practice membership is optional for non-formal sector workers (the bulk of the population). The growth of membership has nevertheless been impressive – with card-holders rising from 6.6% of the population in 2005 to 45% three years later in 2008 (figure 1). This compares very favourably with many other schemes – in Tanzania, for example, total enrolment in the new SHI Benefit scheme is reported to be covering less than 1% of the population [10].

The variation across regions is high, however, ranging from 13% membership in Central Region to 70% in Upper West in 2008. These variations relate in part to previous patterns of health insurance membership (Brong Ahafo, for example, had many schemes in operation prior to the NHIS being established and remains a 'high performer'). Greater Accra has a relatively low membership (32%), related perhaps to greater difficulties enrolling an urban population and a higher ability to pay out-of-pocket for care.

The NHIA does not break down its card-holders by category of member but does provide this information for 'registrants' (a larger group comprising those who have expressed interest in joining, and who have paid some or all of their premia, as well as those who are actually holding valid cards). The evolution in registrants is shown in Table 2. It indicates which groups have fuelled the growth in membership: the formal sector, as would be expected, has not increased much over the past three years and only comprises 3% of registrants in 2008. By contrast, informal sector registrants have grown from 3% to 16% of the population. Another category which has seen a large increase has been the children of members (this group is exempt), which grew from 8% to 27%. The indigent, however, fell from just under 4% of the population to 1% in 2008.

**Table 2:** NHIS registrants, by category, 2006 & 2008

	2005		2008	
**Membership categories**	**Number of registrants**	**Proportion of total population**	**Number of registrants**	**Proportion of total population**

Formal sector	468,092	2.24%	811,567	3%
Informal sector	615,450	2.94%	3,727,454	16%
Paying members	1,083,542	5.18%	4,539,021	19.25%
Pensioners	43,208	0.21%	71,147	0.30%
Children	1,751,175	8.37%	6,305,727	27%
70+	266,421	1.27%	816,956	4%
Indigent	790,078	3.77%	302,979	1%
Pregnant women			432,728	2%
Overall exempt	2,850,882	13.62%	7,929,537	34%
Total	3,934,424	18.79%	12,468,558	54%
% of registrants paying	28%		36%	

*Source*: calculated based on data from annual health sector reviews for 2005 and 2008

The vast majority of NHIS registrants, in 2005 and in 2008, are exempt from making any financial contribution. In this context, it is less surprising that the rate of growth of membership has been so high. In 2005 28% of registrants were contributors (via payroll or informal sector premia). This had risen to 36% in 2008, but this is still only around a third of the total. Large population groups are entitled to free care and this entitlement is being extended. In July 2008 all pregnant women were offered a free annual membership. In 2009 there are plans to extend this to all children (decoupling them from parental membership, which was required in the original Act). While this is positive from a universal coverage perspective, it does present challenges for sustainability (discussed below), and also adds to questions of the extent to which the NHIS is a contributions-based insurance model (Table 3).

**Table 3:** Is the Ghana NHIS a social health insurance scheme?

Key criteria	How the NHIA performs
Is legislated by government and requires regular, compulsory contributions by specified population groups (usually initially covering those in formal employment and their dependants, and then gradually extending to other groups)	The NHIA meets these criteria to some extent, but rather than building up coverage of non-formal groups over time, it has built those in from the start, funded from large tax subsidies. Only around one-third of members have made any financial contribution. 70% of the funding is tax-based.

Has an income-related contribution schedule (i.e. premiums are calculated according to ability to pay), which is uniform even if the SHI consists of a number of health funds serving as the financing intermediaries for the SHI	The NHIA payments are only income-related for the 3% of the population which are formal sector members. For informal members, there is a flat rate premium per person.

Has a standardized, prescribed minimum benefit package	The NHIS does have a standardized, prescribed minimum benefit package

Source: criteria taken from [11]

### Equity

In terms of the equity impact of the switch in funding sources, this has not been investigated in depth. However, generally speaking V.A.T.-based taxation (which is the main funding source for the NHIS) is regressive, as poorer households often have a limited ability to reduce their consumption of taxed goods. By comparison, the general taxation (based on personal and other taxes) which it has substituted for is mildly progressive in Ghana [12].

In relation to benefits, although membership of the NHIS should be universal, in practice there are many barriers to joining – economic, geographic, organizational, and cultural – and so membership remains partial and in some ways skewed against marginalised groups.

While the premium for informal sector members is low (around $5 per person per year), there is also an enrolment fee ($1.5 for all except pregnant women), which together are sufficient to deter many on low incomes. In addition, there are many living remotely who do not have easy access to health facilities and therefore may not perceive the benefits of membership. Analysis of data collected in two districts in 2007 found that while higher economic status affected enrolment (positively) [13], renewal of membership was not affected by economic status, but was affected by location – 88% of urban members said that they were willing to renew, compared with 57% of rural residents [14].

In addition, in the new insurance-financed system, money follows infrastructure – as the regional director for Greater Accra puts it in her annual report for 2008, a hospital with a laboratory will tend to generate more income than a health centre without one. There will therefore be a tendency (as with IGF previously) for higher level facilities to capture reimbursements disproportionately, and similarly with areas (districts or regions) which have higher levels of infrastructure. In this way, historical imbalances between areas may be reinforced and perpetuated.

While the deterrent to recruiting 'indigents' has been removed (collectors are now paid the same commission, whether they recruit paying or exempt members), the definition of 'indigent' in the 2003 Act is very restrictive. Many district schemes have now asked community groups to identify the poorest for enrolment, but it is not clear how effective this strategy will be. The proportion of the total population with NHIS cards for indigents has dropped from 4% in 2005 to 1% for 2008 (Table 2). This is very low compared with an estimated 28% living under the poverty line, according to 2006 Ghana Living Standard Survey figures.

One of the equity concerns relating to the NHIS is how it has affected the non-insured. When a new Diagnosis-Related Group (DRG) tariff was introduced in 2008 it increased 'cash and carry' prices as much as those paid by the DMHIS. For the non-insured, who are commonly the less well off, this will have increased existing financial barriers to health care. Assessing to what extent the non-insured have been 'squeezed out' of the market is not straightforward, but at the fact that two-thirds of 'internally generated revenue' (IGF – income generated by facilities from user fees and the NHIS) is now generated by the NHIS (see below), while membership is 45% of the population, suggests that the non-insured are using fewer services and/or less expensive services. Whether they are using fewer services than they did prior to the tariff increases would require more in-depth study, but it seems intuitively likely, given rises in user fees.

As the NHIS extends towards full coverage, some of the equity concerns will be reduced. In addition, the current regional membership patterns (with high membership levels in the north, less so in areas such as Greater Accra) suggest that the rich in urban areas may be self-selecting out of the scheme for private care (though this hypothesis would need investigation).

### Impact of NHIS on members

#### Financial protection

There has been little research to date on the impact of the NHIS in relation to household care seeking and expenditures, particularly as the NHIS has increased in scale. However, one study has compared baseline data in two districts, before the NHIS (in 2004) and after (in 2007) [15]. Its findings suggest that there has been an increase in access to formal care amongst members, as well as a significant decrease in out-of-pocket expenditure. However, there was no difference in use of maternal care (ANC, deliveries or caesareans) between the intervention and control group, which is an unexpected finding. In addition, the study showed that enrolment in the NHIS remained pro-rich.

Reports of informal payments were rare in the years before the NHIS, with user fee collection closely controlled at health facility level [7]. In the past year, reports of informal payments to health workers have grown. Examples of reported informal payments by clients include:

○ Charging for services out-of-hours

○ Asking patients to pay for drugs which are said not to be in stock

○ Asking patients to pay for 'better' drugs, said to be not provided under the NHIS

One of the factors may be the increased workload for staff resulting from the NHIS – they have experienced a growth in work without any compensation (except for midwives, who do get a small allowance per delivery, according to key informants, at least in some areas). Consequently, they may feel justified in charging small amounts for what they see as 'extra' services. Another factor may be the delays in reimbursement, discussed below.

There are no general data on how household out-of-pocket payments have changed over the last few years in Ghana, though a planned National Health Accounts should illuminate this.

#### Utilisation of services

**Figure 2:**
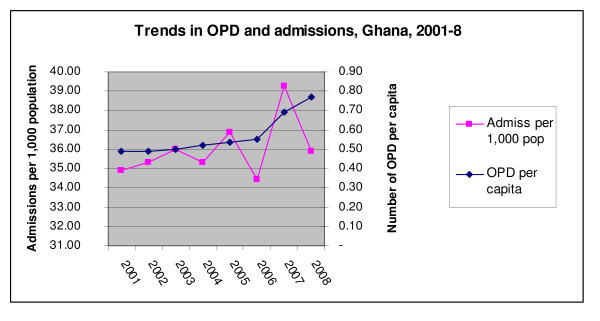
**Trends in OPD and admissions, Ghana, 2001–8**. *Source*: Annual sector review, 2008.

While there is no public information on trends in out-patient (OPD) services by insured patients specifically, OPD use for the population as a whole shows a marked increase from 2005 onward, compared to stable (low) use before (Figure 2). The timing and pattern correlates with growth in NHIS membership, indicating that the NHIS has indeed increased service use. According to an ILO paper of 2006, utilisation for the insured was then at around 0.9 OPD per capita – almost twice the non-insured (then at 0.49 visits per capita) [16]. It is interesting to note however that overall admissions have been stable over the past few years, which is unexpected.

In 2009 the Network of Mutual Health Organisations estimated an average of 1.4–1.5 visits per card holder per year, indicating that there has been the expected growth in service use by members.

### Financial sustainability

#### The emergence of cash flow problems

In the first two years of operation, there was no evidence of cash flow problems in the NHIS, as contributions had built up in the two years prior to operation (from SSNIT contributors) and membership numbers (and therefore claims) were low. In 2008, however, some major cash flow problems developed. As of the end of 2008, around $34 million was owing to health facilities, according to the Ministry of Health financial statement (almost all of this due to unpaid NHIS claims). This was equivalent to 3–4 months' worth of total IGF.

Another symptom of the emerging problem was the growth in payments to 'distressed schemes' (part of the NHIA's reinsurance function). At the end of 2008, GHc 8.32 million was paid out by the NHIA under this budget line (as against a budget of GHc 5 million for the year), rising to just under GHc 30 million in the first quarter of 2009, according to a presentation made by the NHIA Chief Executive in March 2009. This indicates both problems of budgeting and planning, and a growing gap between subsidies to schemes and the costs which they face. Although schemes can apply for 're-assurance', many may be reluctant to do so, partly because of the administrative hurdles involved, but also because of the presumed taint of 'mismanaging' the funds.

The DMHIS are heavily dependent on the subsidies they receive from the NHIA, which provides some 80–90% of their revenue. The original setting of the subsidy amount per member (which has since been adjusted upward by a small amount each year) was apparently based on the premia used by the pre-existing mutual health organisations. However, these were offering very limited packages at the time, so could not have provided an accurate basis for such broad coverage as is offered by the NHIS. In 2008, the fixed transfer per exempted member was GHc 14. Overall figures for claims and expenditure are lacking to assess the adequacy of this subsidy, but in the case of pregnant women, the NHIA tariff for ANC, normal facility delivery and PNC at the lowest level of facility would cost just over GHc 14. Any additional complication, illness during pregnancy or seeking care at higher levels would therefore push the cost over the subsidy level.

Another symptom of the cash short-fall at the district level is the fact that most schemes which used to up-front payments of 40% or so of claims to providers (while claims were being checked) have stopped the practice in 2008. Some scheme managers report that while they had surplus to invest in 2007, there was none in 2008.

#### Underlying challenges to financial sustainability

Underlying the cash flow difficulties now emerging are some fundamental design issues which threaten the long-term sustainability of the NHIS. These are summarised in Table 4.

**Table 4:** Summary of challenges to financial sustainability in NHIS

Dimension	Current challenges
Funding sources	Majority of income grows with growth in consumption, not with membership. Very low premia for informal sector in relation to cost of care.

Benefits package	Benefits package comprises an estimated 95% of all treatments in Ghana, with no limit to consumption.

Coverage	Large proportion of population is exempt and these categories continue to grow Membership is growing and with a growing rate of utilisation by members

Payment systems	Prices have risen with new DRG payment system Drug costs additional – incentive to over-prescribe Anecdotal evidence of 'tariff creep' and gaming by providers Reported increase in fraudulent claims Increasing role of private sector (increases access but also raises costs)

Cost-control	No co-payments Gate-keeping not effective – patients self-refer to secondary hospitals and tertiary ones use their polyclinics as an entry point into specialist care

Monitoring	Poor monitoring and control systems within the NHIS, although a new IT system is being introduced which may improve the situation

While in a 'normal' insurance system, increased membership would bring in increased income from premia, in the NHIS, the income is largely de-coupled – 90–95%, according to the CEO in 2008, coming from SSNIT and the VAT levy. The bulk of its income will therefore grow with national income rather than membership numbers. GDP growth (6.2% in 2008) is below the rate of growth of membership (from 36% in 2007 to 45% of valid card holders in 2008). This means that the more successful the NHIS (in terms of coverage), the greater the risk of financial difficulties.

In 2008 a new tariff structure was introduced by the NHIA, based on a DRG system (paying per episode of care, according to disease groups, but also differentiated by level of care and sector). This replaced the previous payment system which was based on fees for service. Although financial data is not yet available from the NHIA to analyse the overall impact of the new tariffs, scrutiny of selected health facilities' claims shows an immediate jump in NHIS claims, sometimes a doubling within the month of the new tariff being introduced.

Drug costs are currently billed separately on top of the fixed DRG payment per episode, and it is reported by the NHIA that the number of drugs per prescription have increased, from 4.5 in 2004 to 6 now, with some more expensive drugs being particularly favoured by some doctors.

Another common result of the introduction of DRG systems is 'tariff creep' – shifting to diagnoses which attract a higher tariff – which is being reported by NHIA informants. ('We don't get simple malaria cases any more – all malaria is complicated'.)

In addition to the increase in tariffs and increase in members, there has been an increase in utilisation of services by members, which is the expected result of any reduction in financial barriers to care. While this is a positive development (OPD per capita visits remain under the expected norm), it is also something to monitor carefully, in terms of the implications for cash flows and, ultimately, sustainability. Increased utilisation of curative care is not self-evidently positive and care patterns can be distorted by provider interests and also unequal access by different groups. In addition, improving the quality of care is critical to realising health gains from increased utilisation.

The poor gate-keeping in the health system, which is a general issue in Ghana, not limited to care provided under the NHIS, also raises prices, as it means people frequent higher level facilities more, which results in higher reimbursement per episode.

An increase in accredited providers widens access, which is positive, but also has an impact on the cash flow of the NHIS. In 2008, there were 1,551 accredited private providers, providing one-third of all services reimbursed by the NHIS, according to a recent report [17]. Given that the tariff for private providers is higher (and consumers in urban areas have no price disincentive to visiting them), this is likely to be another driver of cost escalation.

There is anecdotal evidence of various types of fraud (against schemes but also, some allege, by and with schemes). It is not easy to assess their scale, not least because some mis-billing reflects lack of understanding of the new tariff. The shift from fee for service, which health facilities are accustomed to, to a DRG-based payment system is not simple. The payment is per illness episode, but the definition of episodes, and the rules about return visits (designed to control costs) are quite complex for providers to follow. In one region visited, an estimated 20–25% of claims presented were rejected, for a variety of reasons.

### Purchasing

Given that the NHIS is largely tax-funded, it may be argued that its main value-added lies not so much in additional resource generation as in creating an independent mechanism for purchasing services in a way which is more responsive than the traditional budget channels. That capacity may develop over time. At present, the NHIS faces constraints in even managing claims effectively, never mind acting as an active purchaser. Its information systems are rudimentary, though there are hopes that a new IT system will help with the transmission of more useful management information from schemes to national level. Currently the IT system is facing its own teething problems.

As the NHIS is pushing up consumption of health care, it is obviously critical that measures are put in place to ensure that the health care provided is appropriate and effective. As one key informant put it: 'We want good health outcomes – not just to inject money!' This will involve investment on the provider side – investment in quality assurance systems, which are currently underfunded – as well as the development of an accreditation system, which has recently been piloted by the NHIA [18].

### Overall impact on health system

The role of the NHIS has changed significantly over the past four years. Its original intention was to replace user fees and so reduce financial barriers to health care, inequity and health-related poverty. This is now changing, with a greater proportion of health care being funded through the NHIS channel. While user fees only constituted around 12–14% of the overall resource envelope in the first half of the decade, the NHIS is now estimated to contribute 41% of overall revenue (according to the Medium Term Expenditure Framework for 2009).

**Figure 3:**
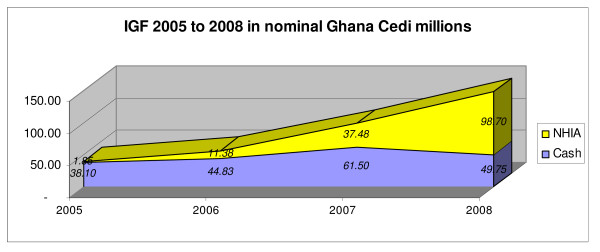
**'Internally generated funds' in Ghana, 2005–8: NHIS and 'cash and carry'**. *Source*: [19].

Initially it added to, rather than supplementing 'cash and carry' IGF: only in 2008 did the cash and carry component start to reduce (Figure 3).

Instead of merely covering some of the recurrent non-salary costs of services, as user fees used to, it is now gradually expanding to fund other functions. In the guidelines for the 2009 budget, items 3 (service costs) and item 2 (administration) are either reduced or cut entirely for facilities which generate income, and hospitals are directed to set aside 10% of IGF revenue for replacement of equipment and minor rehabilitation of infrastructure. IGF revenues are also used to pay staff. The overall need to control growth in 'personal emoluments' (wages and allowances) may be circumvented by the fact that institutions are increasingly able to hire 'casual' staff, paid from IGF. The trend towards funding the full cost of curative care from the NHIS poses a risk, if the management of the NHIF is not sustainably managed.

**Figure 4:**
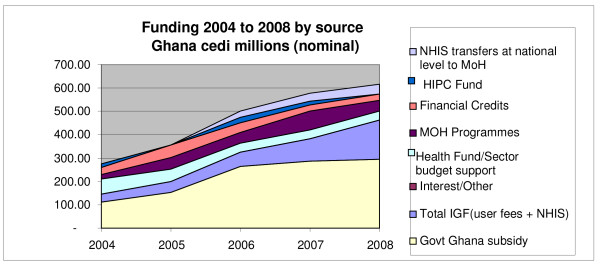
**Overall health sector funding, 2004–2008, by source**. *Source*: MoH Financial Statements, 2004–2008.

Many regard the NHIS as additional funding, but the reality is that its growth has coincided with a clear levelling off (i.e. reduction in real terms) of the core government subsidy to health (Figure 4). The NHIS is included in the sector budget, and what it adds is evidently adjusted for by the Ministry of Finance. Looking at longer term trends, the proportion of public spending allocation to health has grown from 8.4% at the start of the first SWAp in 1997 to hitting the Abuja target of 15% in 2005 (prior to the NHIS start-up). Since then, the allocation to health, including the NHIS funding, has remained around that level (the proportion was 14.9% in 2008; the allocation for 2009 is 15%). The total annual expenditure on health per capita (including government funds, donors funds, NHIS contributions and user payments to public facilities) was $25 in 2006, dropping slightly to $23 in 2007 and 2008 (the drop partly reflecting changes to the exchange rate).

The financial impact of the NHIS on providers is complex to assess. On one hand, price and activity levels have increased, all of which increase revenue and lower unit costs. On the other hand, reimbursement delays obviously damage cash flow and non-payment causes longer term debts (to suppliers, such as the medical stores and others). At the facility level, how have the revenues from the NHIS been used (from hospitals down to the lowest levels – the CHPS compounds)? Anecdotally, some have used them to upgrade facilities and pay for maintenance. Others are sitting on balances, perhaps because it is hard to get approval from district managers to release funds. Some reports of overcrowding, particularly in OPD, have been reported but it is not known if this is a general problem or specific to some facilities. It is important to look in more detail at how facility activities have been affected.

The regions with higher membership are particularly dependent on NHIS reimbursements – in Northern region, for example, nearly 90% of IGF in 2008 came from the NHIS. In one district hospital, visited at random, government 'core budget' funds provided 6% of total revenue for 2008; programme funding 4% and IGF 90%.

A separate concern is the balance between preventive and curative services. At present, those facilities generating revenue from the NHIS are becoming increasingly financially independent, while funds for public health activities, while not falling, are stagnant. This changes the power balance between hospitals, in particular, and health managers at district, regional and national levels. Ensuring that this does not contribute to increased health sector fragmentation requires careful thought and action. While there is some evidence that facility funds at the sub-district level are benefiting the wider district (relieving the need for district managers to pay for fuel for outreach activities by the health centres, for example), there is no evidence of any financial redistribution from hospitals to health administrations.

The current payment system risks creating 'perverse incentives' to provide more curative and less preventive health care. In other countries, 'payment for performance' systems tend to incentivise public health activities. If the NHIA remains a significant funding channel for health care in Ghana, some thought might be given to modifying it to cover preventive care. This would not only benefit the health of Ghanaians but also save the NHIS money. This was noted in a recent study, which estimated the cost savings which could be generated by including family planning in the NHIS benefits package (Banking on Health 2008). Clients currently face a small charge for family planning commodities and services, which seems at odds with the policy of providing free delivery care through the NHIS.

### Governance and accountability

Looking at the original Act and Legislative Instrument, there was some ambiguity in the accountability arrangements for the NHIS, which has led to a period of institutional conflict. Some articles refer to the role of the Minister – for example, in Article 2.2.h, it refers to the NHIA as making 'proposals to the Minister for the formulation of policies on health insurance', which implies that the Minister exercises oversight. Others refer to the role of Parliament, for example, in approving allocation of funds (78.1.c). Others again reflect the role of the President, for example, in appointing the Executive Secretary of the NHIA (92.1). The Chief Executive has taken this to mean that he is ultimately responsible to the President, rather than to the Minister of Health, and cooperation and information sharing between the NHIA and the MoH has not been strong.

In addition, the corporate culture of the NHIA has not been open until now, with routine data being treated as confidential material. This has reinforced a sense of lack of transparency, and of a fragmented sector, not working together. Annual and financial reports have not been circulated in a timely way. A business plan was developed some years ago but has still not been shared. Very little information on the situation of the DMHIS filters up to the national level. There appears to be no concerted plan for monitoring and evaluation.

In addition to issues of governance and transparency, there are debates about the structure of the NHIS itself. The NHIA is proposing some fundamental changes to the way in which the NHIS works: it presented amendments to LI 1809 to Cabinet in 2008, but these would now have to be re-presented under the new government. The objective was to merge the district schemes. This offers advantages in terms of creating a single risk pool and also potential efficiencies in terms of processing claims in ten regional centres (rather than 145 district schemes). Set against that is the loss of local accountability and potential for local mobilisation which was a feature of the original 2003 Act. This is a very important political decision which should receive wider public discussion in Ghana.

## Discussion

The NHIS is a very bold national experiment, which is now, like the banking system, 'too big to fail'. It has already undergone a number of evolutions and will almost certainly embark on more. How can it be judged so far in terms of its overall contribution? Given the tendency of reforms to drift over time and to be influenced by wider political and stakeholder interests, other analysts have advocated benchmarking social health insurance against its original objectives [11]. In the case of Ghana, the original objectives were essentially political, but focussed on removing financial barriers for households to access health care. Against these objectives, it has performed relatively well – covering a substantial proportion of households in a relatively short period of time, and reducing household expenditure, according to the one impact evaluation conducted to date (though it should be noted that this study only focussed on a small number of areas and that its data collection did not extend into the recent period in which facility reimbursements have been delayed, which may have had a knock-on effect on household payments).

More generally, SHI typically has two main goals – to increase revenues and to improve the equity and efficiency of the health system [11]. In relation to these goals, Ghana's achievements are less clear-cut. The increased funding through the NHIS appears to have substituted for other public revenue. In terms of equity, there remain pro-rich biases in membership of the NHIS, and negative impacts on non-members, who face higher payments for services. NHIS members, meanwhile, enjoy the double public subsidy of the use of the NHIL levy as well as benefiting from increased access to prior public subsidies to the system (for salaries, investment costs etc.). Of course, the pro-rich bias of membership has to be compared with previous patterns of access to care. There are few recent benefits incidence studies for Ghana, but one conducted in the early 1990s showed that public funding for hospitals and in totality was weakly pro-rich but, at the health centre level, use appeared to be proportional to sickness across all groups [20]. The overall effect of the NHIS is therefore likely to have exacerbated a previously weakly pro-rich utilisation of public funds.

In relation to horizontal equity, the design of the NHIS is positive, with equal contributions across the country, risk pooling between schemes (through reinsurance) and the offer of a standard package of care, although the ability to physically access care will determine the extent to which these benefits are realised. While geographical inequality existed before the NHIS, it risks being exacerbated by it, inasmuch as those living in proximity to facilities can, by joining, reduce the financial barriers they faced and increase their utilisation.

The evidence on efficiency is ambiguous too – while rising utilisation could increase efficiency by reducing unit costs, there are also efficiency losses through the additional revenue collection costs (NHIL and premia), the NHIS overheads, the increase in supply-induced demand for NHIS clients, the increased ability to self-refer to higher level facilities, and the apparent shift in funding from public health/preventive to curative activities (an area which requires more in-depth analysis).

Another traditional argument for SHI is that it increases the responsiveness of services [21], as members have a stronger entitlement than mere tax-paying consumers. There is no evidence yet that this is happening in Ghana, though the efforts at establishing systems for accreditation and complaints channels for clients are attempting to shift the system in that direction. The NHIA is accountable to Parliament, just as the Ministry of Health is. Its accountability to members is not very apparent and the plan to merge the DMHIS will undermine whatever local accountability systems exist at district level (these are very variable in practice – strong in some areas but close to non-existent in others).

With its heavy reliance on tax funding and its inclusion of major population groups whether they have contributed or not, the NHIS in many ways resembles a tax-funded universal health care system more than a SHI one. The main distinction is the third-party payment channel for curative care. Whether the substantial transaction costs of setting up this parallel system are justified (adding to the plethora of agencies operating in the health sector in Ghana) will depend on the extent to which the NHIS is able to act as a cost-effective and responsive purchaser. As noted, its capacity in this respect is currently weak. The switch from traditional budget ceilings to payment per curative episode also creates a strong incentive for cost escalation, which threatens the overall financial sustainability of the NHIS. The current delays in reimbursement are an early sign of the problem. Reforms will be needed to square the circle of a broad and open-ended benefits package, wide population coverage and limited funding.

## Conclusion

Establishing a social health insurance system takes time – countries like Germany have taken a century to reach universal coverage through this route. It is therefore early days for the NHIS, which was only launched in 2005, and further evolution is inevitable. However, it is important that it is carefully monitored and challenges facing it openly debated. The lessons from Ghana should also inform other experiments in the region.

Some trade-offs will be necessary between the existing wide benefits package with no co-payments and the laudable desire to reach universal coverage. The overall resource envelope for health is likely to be stable rather than increasing over the medium-term. The NHIS has not leveraged additional resources for health care in Ghana but has changed the funding channels. The jury is still out on how that has affected equity and efficiency in the system. In addition, there are pressing challenges to financial sustainability which will require more cost control measures to be put in place. In the longer term, the investment costs in the NHIS will only be justified if it is able to increase the cost-effectiveness of purchasing and the responsiveness of the system as a whole.

## Competing interests

The authors declare that they have no competing interests.

## Authors' contributions

SW prepared the first draft of the paper. BG contributed to analysis and refining of the draft. Both authors read and approved the final version.

## Pre-publication history

The pre-publication history for this paper can be accessed here:

http://www.biomedcentral.com/1472-698X/9/20/prepub
